# Intracranial aneurysm segmentation with nnU-net: utilizing loss functions and automated vessel extraction

**DOI:** 10.20517/2574-1209.2025.42

**Published:** 2025-12-09

**Authors:** Maysam Orouskhani, Negar Firoozeh, Huayu Wang, Yan Wang, Hanrui Shi, Weijing Li, Beibei Sun, Jianjian Zhang, Xiao Li, Huilin Zhao, Mahmud Mossa-Basha, Jenq-Neng Hwang, Chengcheng Zhu

**Affiliations:** 1 Department of Radiology, University of Washington, Seattle, WA 98195, USA.; 2 Department of Electrical and Computer Engineering, University of Washington, Seattle, WA 98195, USA.; 3 Department of Radiology and Biomedical Imaging, University of California San Francisco, San Francisco, CA 94143, USA.; 4 Department of Radiology, Ren Ji Hospital, Shanghai Jiao Tong University School of Medicine, Shanghai 200001, China.

**Keywords:** Aneurysm segmentation, nnU-Net, TOF-MRA

## Abstract

**Aim:**

Intracranial aneurysms pose significant challenges in diagnosis and treatment, emphasizing the need for accurate segmentation methods to assist clinicians in their management. In this paper, we present a novel approach for segmenting intracranial aneurysms using three-dimensional time-of-flight magnetic resonance angiography (TOF-MRA) images and the no-new-U-net framework. We aim to improve segmentation accuracy and efficiency through the integration of hybrid loss functions and additional vessel information.

**Methods:**

The model was conducted on Aneurysm Detection And SegMentation (ADAM) and Renji Hospital (RENJI) datasets. The TOF-MRA ADAM dataset contains data from 113 cases, where 89 have at least one aneurysm with a median maximum diameter of 3.6 mm and range from 1.0 to 15.9 mm. The RENJI private TOF-MRA dataset comprises 213 cases including both ruptured and unruptured aneurysms with a median maximum diameter of 9.35 mm (range: 1.25-37.58 mm). We optimized the segmentation model by exploring hybrid loss functions that combine distribution-based and region-based losses to effectively delineate intricate aneurysm structures. Additionally, we incorporated vessel information as a region of interest using an automatic vessel segmentation algorithm to enhance the model's focus on critical regions. The model was trained on multi-modality data, including both vessel-enhanced and original images, to capture complementary information and improve segmentation accuracy.

**Results:**

Extensive simulations on both the ADAM dataset and a private RENJI dataset demonstrate the effectiveness of our approach. The best-performing loss function yielded significant improvements in the Dice coefficient (0.72 and 0.54) and Sensitivity (0.69 and 0.53) on the RENJI and ADAM datasets, respectively.

**Conclusions:**

The proposed method offers a promising solution for accurately segmenting intracranial aneurysms, showcasing superior performance compared to existing approaches. By integrating hybrid loss functions and vessel information, we enhance the model's ability to delineate intricate aneurysm structures, contributing to improved diagnosis and treatment planning for patients with intracranial aneurysms.

## INTRODUCTION

**Figure 1.**
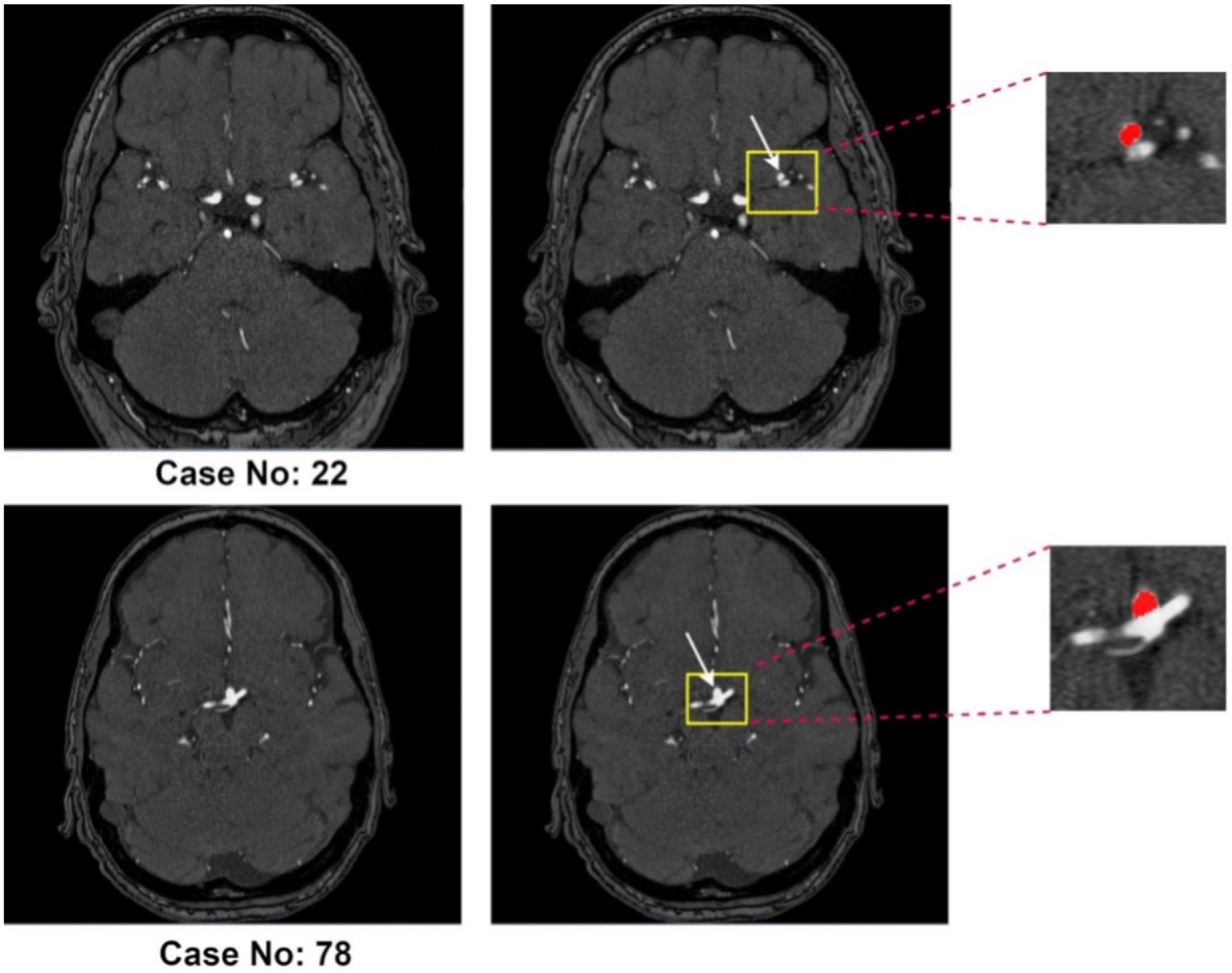
Representative 3D TOF-MRA cases from the ADAM dataset. Case No. 22 (top row) and Case No. 78 (bottom row) both include intracranial aneurysms (highlighted in yellow boxes and magnified on the right, with the aneurysm mask shown in red).

Approximately 3% of the global population harbors an unruptured intracranial aneurysm (UIA)^[1]^. In high-risk groups, particularly those with a positive family history of aneurysmal subarachnoid hemorrhage (aSAH), the prevalence increases to about 10%^[2]^. Rupture of an intracranial aneurysm leads to aSAH, a severe form of stroke associated with high morbidity and mortality: roughly one-third of patients do not survive and another third are left with permanent, life-altering disabilities^[3]^. Early and accurate detection of UIAs during screening is therefore crucial for assessing rupture risk and guiding management. Evidence from large observational cohorts indicates that factors such as aneurysm size, location, and morphology - including irregular shape and daughter sacs - are associated with increased rupture risk^[4-6]^. Current practice generally recommends preventive treatment for aneurysms with high predicted rupture risk, while lower-risk aneurysms are managed with regular imaging follow-up to monitor for growth, which itself is a strong predictor of rupture^[4,7]^. This approach allows clinicians to make more informed treatment decisions based on individualized risk assessment. With the increasing availability and quality of cerebral vascular imaging, incidentally discovered UIAs have become more frequent, leading to a greater need for reliable follow-up imaging^[8]^. The most commonly used techniques for UIA monitoring include contrast-enhanced computed tomography angiography (CTA) and non-contrast three-dimensional (3D) time-of-flight magnetic resonance angiography (TOF-MRA), with TOF-MRA being particularly suitable for routine surveillance because it avoids intravenous contrast agents and ionizing radiation^[9-11]^. Figure 1 illustrates two representative TOF-MRA cases with annotated intracranial aneurysms from the Aneurysm Detection And SegMentation (ADAM) dataset (Case 22 and Case 78).

Accurate segmentation of intracranial aneurysms may play an important role in clinical practice for several reasons. First, precise localization and measurement of aneurysms are enabled, providing essential information for treatment planning and monitoring disease progression. Second, the assessment of aneurysm morphology is facilitated, and the prediction of the risk of rupture is aided by segmentation. Size, shape, and location are crucial factors contributing to rupture risk stratification, guiding the selection of appropriate interventions. Moreover, detailed analysis of hemodynamic parameters, such as blood flow patterns, wall shear stress, and pressure distribution, is facilitated by aneurysm segmentation, which is important in understanding aneurysm pathophysiology. However, accurate segmentation of intracranial aneurysms poses several challenges. The complex and irregular shape of aneurysms, coupled with their proximity to delicate structures and the presence of noise in medical images, makes manual segmentation labor-intensive, time-consuming, and prone to inter-observer variability. Therefore, there is a growing need for automated and efficient segmentation methods that can aid clinicians in accurately identifying and analyzing intracranial aneurysms.

In recent years, the emergence of deep learning techniques as a powerful tool in medical image segmentation has revolutionized the field by harnessing the vast amount of available data and computational resources^[6-8]^. Remarkable performance in various medical imaging tasks, including segmentation, has been demonstrated by convolutional neural networks (CNNs). By automatically learning hierarchical features from input data, these models capture complex patterns and contextual information, enabling accurate and efficient segmentation of intracranial aneurysms. Several Magnetic Resonance (MR)-based deep learning models for automatic segmentation have been proposed^[9-11]^. While some works have introduced new machine learning strategies to improve segmentation performance^[12-17]^, others have focused on incorporating vessel information.

The integration of vessel information has shown promise because blood vessels provide a valuable anatomical context closely associated with aneurysms. Prior vessel-aware approaches typically rely on vessel segmentation algorithms to extract vascular structures and use them as regions of interest (ROIs) or auxiliary guidance. However, these methods face several limitations: (i) vessel segmentation errors - particularly in thin, tortuous, or low-contrast vessels - can propagate to aneurysm prediction; (ii) most studies rely on binary vessel masks, which neglect complementary intensity and contextual cues; and (iii) small aneurysms adjacent to large vessels often remain difficult to detect due to class imbalance and lossfunction insensitivity. These challenges limit robustness and sensitivity, especially for subtle or complex aneurysm cases.

In this paper, we propose a novel approach that directly addresses these limitations using 3D TOF-MRA images with the nnU-Net (“no-new-Net”) pipeline. The main contributions of this work are as follows:

### - Hybrid loss functions:

We investigate combinations of distribution-based and region-based losses to balance sensitivity to small aneurysms with accurate boundary delineation. This directly mitigates the insensitivity of prior methods to small lesions and class imbalance.

### - Automatic vessel segmentation as ROI guidance:

To reduce error propagation from imperfect vessel extraction, we design an unsupervised vessel segmentation algorithm that enhances vessel contrast using the Hessian matrix, applies Otsu’s thresholding for separation, and refines results through dilation, erosion, and non-maximum suppression. This yields a more reliable ROI that guides the model’s attention to aneurysm-relevant regions.

### - Multi-modality strategy:

Instead of treating vessel masks as binary guidance, we generate two complementary modalities - the cropped ROI and a vessel-enhanced image multiplied by the Hessian filter output. By training the model on both, we enable it to capture richer contextual and intensity information, improving robustness across aneurysm sizes and locations.

The effectiveness of our approach is validated on the ADAM dataset and a private RENJI dataset. Our method achieves superior performance compared to existing techniques, demonstrating that hybrid losses and vessel-enhanced multi-modality inputs can overcome key limitations of prior vessel-aware segmentation work.

## METHODS

### Datasets

#### Adam dataset

ADAM, the dataset provided by the Aneurysm Detection and Segmentation Challenge^[18]^, contains data from 113 cases, where 89 have at least one aneurysm. The dataset contains TOF-MRA images and manually created references (masks) of the images and locations of aneurysms in text files. All images were preprocessed using correction to adjust the bias field.

The UIAs had a median maximum diameter of 3.6 mm and ranged from 1.0-15.9 mm. Additionally, 30% (*N* = 27) of the scans contained multiple UIAs, and all cases involved saccular aneurysms except for 2 cases.

#### Renji dataset

RENJI, a private dataset, comprises 213 cases including both ruptured and unruptured aneurysms from RENJI Hospital in China. The aneurysms had a median maximum diameter of 9.35 mm and ranged from 1.25 to 37.58 mm. Among these cases, 12% (*N* = 25) feature more than one aneurysm, while 91% (*N* = 198) consist of at least one saccular aneurysm, and 25% (*N* = 55) include at least one fusiform aneurysm. Notably, 51% (*N* = 108) include at least one aneurysm exhibiting internal dark signal regions. Within this dataset, there are nine ruptured aneurysms (the dataset was obtained with the approval of the Institutional Review Board to ensure ethical compliance). Table 1 summarizes the characteristics of the ADAM and RENJI datasets, which encompass the distribution of cases based on aneurysm size, the influence of dark signal, presence of multiple aneurysms, and the shape of aneurysms.

### Methodology

Our approach to aneurysm segmentation involved utilizing nnU-Net^[19]^ as the foundational framework. To optimize results, we explored two key ideas. First, we proposed various hybrid loss functions to analyze their individual effects on segmentation performance. Recognizing the absence of a one-size-fits-all optimal loss function for medical image segmentation, we systematically evaluated the impact of different losses on the model's effectiveness. Second, we addressed the challenge of irrelevant features in high-resolution 3D magnetic resonance angiography (MRA) data. Initially, manual cropping was applied, but variations in the ROI among different images prompted the development of a dynamic, traditional image processing method. This method detects and enhances brain vessels, allowing us to dynamically crop each image based on the true coordinates of the vessels. By focusing attention on the vessels, we effectively filter out irrelevant features, aiding in the accurate detection of aneurysms associated with the vessels. Finally, we incorporated multi-modality imaging by training the model with both the original image and a Vessel Enhanced-Attention (VEA) image. This approach involves enhancing the vessel region in the image and performing a dot multiplication with the original image, resulting in improved model training with a heightened focus on critical areas.

#### nnU-net

In this study, we used the 3D full-resolution nnU-Net as the baseline model for intracranial aneurysm segmentation^[19]^. Following its default pipeline, our implementation included automatic cropping of the non-informative background, Z-score intensity normalization of input volumes, and standard data augmentations such as rotations, scaling, and mirroring.

#### Various losses

Modern end-to-end learning algorithms, such as deep neural networks (DNNs), can automatically extract features from high-dimensional images. This capability makes them a powerful tool for accurately segmenting aneurysms, a crucial aspect of the quantitative analysis of aneurysms. However, the success of a DNN relies heavily on its loss function, which measures the dissimilarity between the ground truth and the predicted segmentation. While a single loss function addresses certain aspects of optimal similarity embedding, compound loss functions are often preferred.

Although cross-entropy and Dice loss have become widely used in general medical image segmentation, recent advancements have introduced new loss functions to capture diverse features of embedding. This is particularly important in addressing challenges such as imbalanced data in aneurysm datasets, where the distribution of classes is unequal. To tackle this issue, we propose a novel compound loss that combines different loss functions from distribution-based and region-based categories. This ensemble of losses enhances the deep model, with the distribution-based loss minimizing dissimilarity between two distributions of embeddings, and the region-based loss maximizing the overlap between ground truth and predicted segmentation. In this paper, we integrate a 3D full-resolution nnU-net with this new compound loss function to capture various aspects of the embedding layer.

In addition to the standard nnU-Net hybrid loss function combining Dice and cross-entropy, we explore the impact of each loss on the segmentation model using TopK^[20]^, Focal^[21]^, and Tversky^[22]^. These include combinations such as Dice + CE, Dice + TopK, Dice + TopK + CE, Dice + Focal, Dice + TopK + Focal, Dice + TopK + Focal + Tversky + CE, weighted Dice + TopK + CE, and weighted Dice + TopK + Focal + Tversky + CE. Each loss function serves a specific purpose: Cross entropy (CE) measures the difference between probability distributions; Dice loss quantifies the overlap between ground truth and segmentation based on per-voxel classification; TopK loss focuses on hard samples during training; Focal loss addresses class imbalance, and Tversky loss is an improvement over Dice loss for handling imbalanced data. The details are indicated in Table 2.

#### Automatic vessel cropping

**Figure 2.**
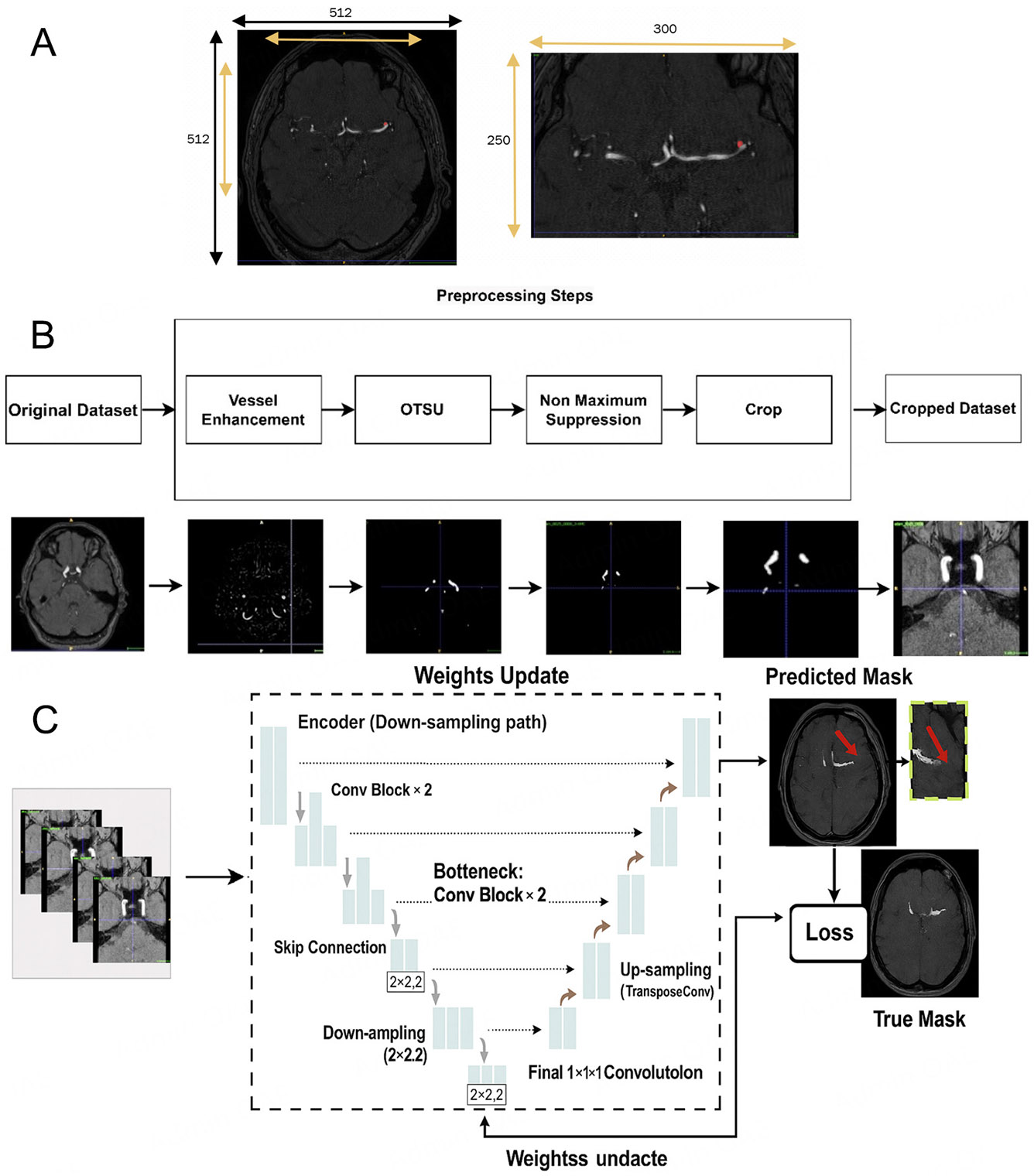
Example of a single 3D TOF-MRA case shown at different stages of our pipeline. (A) Manual cropping; (B) Automatic cropping method with traditional image processing methods: vessel-enhanced image obtained with the multi-scale Hessian filter and final cropped region of interest (ROI) based on the automatic vessel mask, which is then used as input for the aneurysm segmentation network (C).

Cropping is vital for efficiently processing 3D MRA images, allowing focused attention on key ROIs in segmentation tasks. This targeted approach improves segmentation quality, reduces noise, and enhances memory efficiency. However, the cropping strategy must be carefully designed to avoid removing diagnostically important structures. For automatic vessel-based cropping of 3D TOF-MRA images, we employ a multi-step approach integrating vessel enhancement, segmentation, and post-processing. We first apply a multi-scale Hessian filter to enhance tubular structures. The eigenvalues of the Hessian matrix effectively describe local geometric shapes, and their scale-space representation allows us to highlight vessels of varying calibers. We use a Frangi vesselness function as the similarity measure because of its strong performance on cerebrovascular MRA data. Vesselness responses are computed at σ = 0.5, 1.0, 2.0, and 3.0 mm, and the maximum response across scales is retained to capture both small and large vessels. A Gaussian prefilter at σ = 0.5 mm is applied to suppress high-frequency noise before computing the Hessian. Following enhancement, Otsu’s adaptive thresholding is used to segment vessel regions from background tissue. Because the grayscale values of vessels are significantly higher than those of surrounding tissues after enhancement, this step effectively binarizes the image. To reduce false positives, we apply non-maximum component suppression (NMS) to remove disconnected or spurious structures. We also consider the expected connectivity of the circle of Willis to guide suppression of non-maximum components. The final step involves computing the extrema of the vessel mask to define the minimum bounding cube encompassing all vessels. This cube is then applied to both the MRA image and the mask to narrow the input region for aneurysm segmentation, reducing noise and improving inference speed while preserving all vessel regions where aneurysms may occur. Figure 2 illustrates the comprehensive process of these steps. Figure 2A demonstrates the manual cropping, while Figure 2B exhibits the automatic cropping method utilizing traditional image processing techniques. As shown in Figure 2C, the automatically cropped ROI generated by our vessel mask is used as the final input to the nnU-Net segmentation model.

Note about automatic cropping, The cropped images are fed into a 3D U-Net (from nnU-Net) for segmentation. We use 3D convolutional kernels to capture volumetric information. When computing the loss, we calculate the loss function between the corresponding patches and the regions in the ground truth. Figure 2B: After vessel enhancement, we perform Otsu in full 3D rather than slice-by-slice. Specifically, we compute a single global threshold from the histogram of the entire 3D volume and apply it voxel-wise to the Hessian-enhanced image. This approach yields a more consistent binarization across slices and preserves inter-slice continuity of the vessels. For non-maximum suppression, we identify connected components in the binarized 3D mask using 26-voxel (3D) connectivity (i.e., voxels sharing faces, edges, or corners are considered connected). This is more permissive than 6- or 18-connectivity and better reflects the tortuous nature of intracranial vessels. We then discard small isolated components as false positives. In our implementation, any connected component smaller than 500 voxels (approximately 0.5 mm^3^ at our voxel size) is removed. This threshold was chosen empirically based on visual inspection of several cases to retain all plausible intracranial vessels while suppressing noise and nonvascular structures.

#### Multi-modality training

The incorporation of multi-modality in deep learning training confers several attention-related benefits. By integrating diverse information, such as different imaging modalities, the model gains a more comprehensive understanding of the underlying patterns within the data. This enhanced input not only provides a richer context for the model but also allows it to focus on distinctive features present in each modality. The synergistic utilization of multiple modalities enables the model to develop a more robust and nuanced representation of the input, improving its attention to critical details. This approach enhances the model's ability to discern complex relationships, generalize across different aspects of the data, and ultimately boost its overall performance in tasks ranging from classification to segmentation. The multi-modality training strategy thus facilitates a more attentive and context-aware deep learning model.

**Figure 3.**
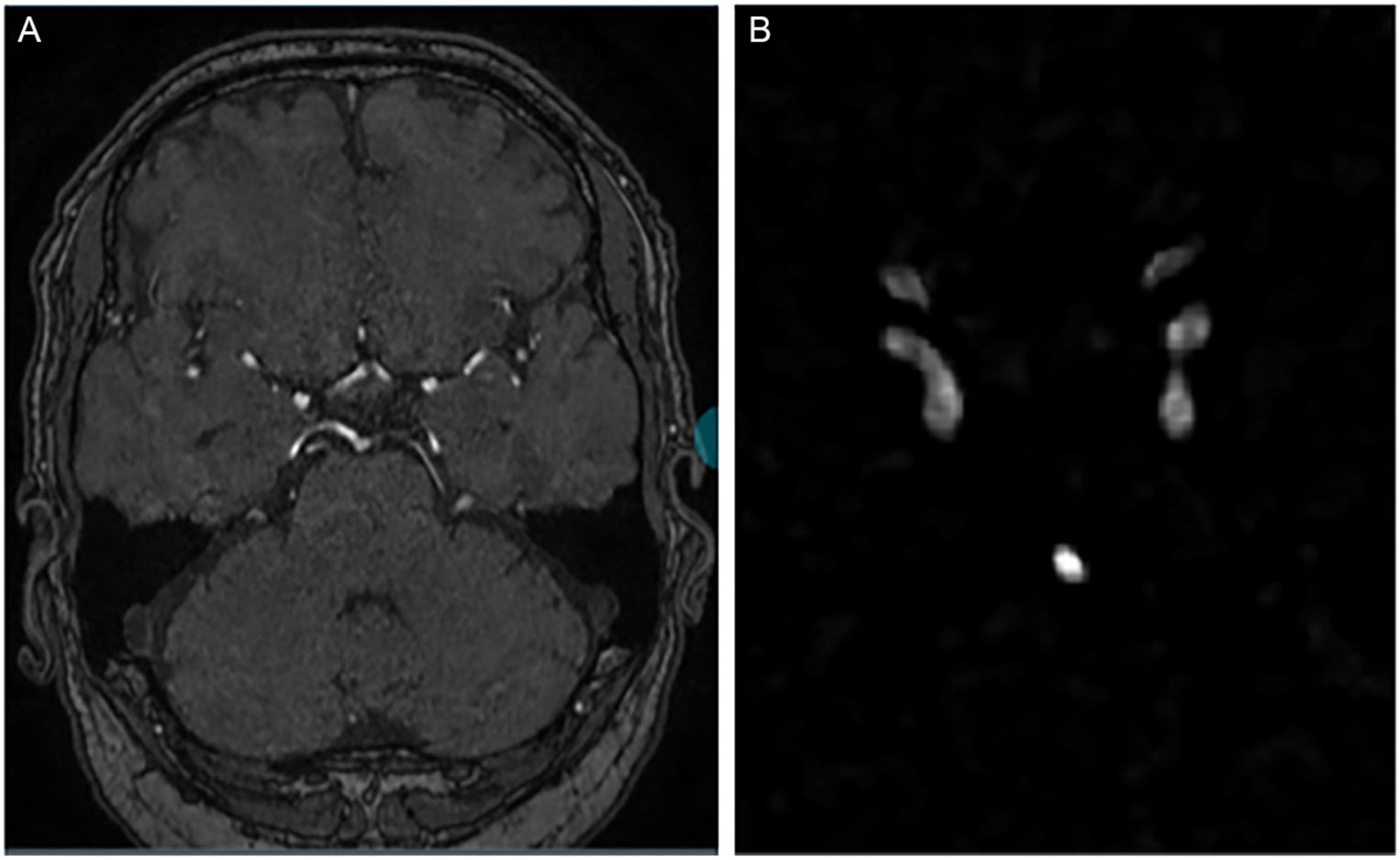
Two modalities for training nnU-Net. (A) The original 3D TOF-MRA, (B) the normalized and enhanced vessel image extracted from the Hessian matrix.

The intricate connection between arterial aneurysms and blood vessels underscores the significance of focusing on vessel-related features for accurate detection. In our pursuit to heighten the network's attention to these critical regions, we adopted a multi-modal approach by incorporating diverse data sources into the input image. After enhancing the image using the Hessian matrix filter, we went a step further to normalize the resulting image, creating a mask that emphasizes vascular structures. We then executed element-wise multiplication between the mask and the original image. This strategic operation not only accentuates the vascular regions but also effectively suppresses non-relevant tissues. By refining the network's attention to these specific anatomical areas, our approach goes beyond traditional methods, laying a foundation for more robust detection of arterial aneurysms. This multi-modal strategy not only bolsters the model's sensitivity to vascular features but also contributes to a more nuanced understanding, ultimately enhancing the precision and reliability of aneurysm detection in medical imaging applications. Figure 3 shows two modalities for training the nnU-Net.

#### Network architecture

Our methodology employs nnU-Net, an automated deep-learning framework specifically designed for medical segmentation tasks. To establish a foundational reference for subsequent modifications, we initialized the use of nnU-Net in its default configuration. The architecture of the neural network follows that of a 3D U-Net at full resolution. This design encompasses both encoding and decoding pathways, with each pathway incorporating five convolutional blocks. Each of these blocks consists of a convolutional layer with 3 × 3 × 3 dimensions. In addition, we incorporated the instance normalization layer and employed the leaky rectified linear unit activation function. Before model input, nnU-Net employs cropping and Z-Score normalization techniques for image preprocessing. We opted for the Stochastic Gradient Descent (SGD) optimization algorithm with an initial learning rate of 0.01, momentum of 0.9, and weight decay of 1 × 10^−4^. A cosine annealing learning-rate schedule with restarts was employed to gradually decrease the learning rate during training and improve convergence stability. The model was trained for 250 epochs using a five-fold cross-validation strategy. All model training was conducted on three RTX-3090 GPUs with a batch size of 2 and a patch size of 256 × 224 × 56. In line with the default configuration of the 3D full-resolution nnU-Net framework, we used instance normalization rather than batch normalization because (i) the small batch size required for 3D patches on GPU memory makes batch-statistics estimation unstable; and (ii) instance normalization is less sensitive to inter-subject intensity variations common in medical imaging, which improves model generalization.

## RESULTS

### Metrics

In assessing the effectiveness of deep learning-based aneurysm segmentation in this study, a pivotal role is played by the utilized evaluation metrics. A comprehensive picture of the model's performance is provided collectively by the Dice coefficient [Dice similarity coefficient (DSC)], Recall, Precision, and 95th percentile Hausdorff distance (HD95). The degree of overlap between the model's predictions and the ground truth is quantified by the DSC, encapsulating the balance between true positives, false positives, and false negatives. The model's ability to accurately identify aneurysm regions is highlighted by Recall, while Precision is defined as the ratio of true positive predictions to the total number of positive predictions, quantifying the correctness of positive predictions. HD95 assesses the maximum distance between the predicted and ground truth segmentation boundaries and calculates the 95th percentile of the Hausdorff distance, offering a robust measure of segmentation accuracy while discounting outliers. Better segmentation results are indicated by lower HD95 values. Together, these metrics form a holistic evaluation framework, facilitating a thorough assessment of the model's suitability for clinical applications in aneurysm detection and localization.

### Experiments

This section presents simulation results for three key ideas conducted on both ADAM and RENJI datasets. First, we showcased the outcomes of training nnU-Net with various loss functions. Following this, we conducted a comparison of segmentation results, evaluating the model's performance with both manually cropped data and vessel-based automatically cropped data. Lastly, we delve into the results of multi-modality training, wherein the model is trained using both the original image and a vessel-enhanced image.

#### Effect of different losses

In this section, we explore the effectiveness of different hybrid losses in training the nnU-Net for aneurysm segmentation in ADAM and RENJI datasets. While we use the hybrid losses including combinations such as Dice + CE, Dice + TopK, and Dice + TopK + CE, the goal is to identify the most effective loss for aneurysm segmentation.

Tables 3 and 4 present the results obtained through the utilization of various loss functions during the training of nnU-Net for aneurysm segmentation in the ADAM and RENJI datasets. Table 3 provides a detailed comparison of performance metrics, including Dice, Sensitivity, and Precision, for each loss function in ADAM. It also offers insights into the mean and standard deviation (SD) values. The analysis of the results in Table 3 reveals the mean and variation across five folds in performance metrics across different hybrid loss functions employed for nnU-Net training in aneurysm segmentation. The Dice coefficient, a key measure of segmentation accuracy, demonstrates the highest mean value for the weighted Dice + Top K + CE combination (0.5467) and the second highest value belongs to Dice + CE, indicating superior overlap between predicted and ground truth regions. Sensitivity, representing the model's ability to identify true positive instances, is highest for weighted Dice + CE + TopK (0.5377) while Dice + CE got the next highest value. Precision, reflecting the ratio of true positive predictions to the total positive predictions, shows the highest mean value for weighted Dice + CE + Top k (0.7118) and weighted Dice + CE + TopK+ Focal + Tversky (0.6911), indicating a high correctness of positive predictions. The variations in these metrics highlight the impact of different loss functions on the model's performance. While the weighted Dice + Top k + CE provided the minimum SD for Dice and Sensitivity, the combination of Dice and Focal obtained the minimum variation in Precision. However, the choice of the optimal hybrid loss depends on specific priorities, such as emphasizing precision over sensitivity or achieving a balanced Dice coefficient. In summary, based on Table 5, the combined use of weighted Dice + CE + TopK achieved the highest performance across all metrics, with Dice + CE securing the second position in terms of ranking.

**Figure 4.**
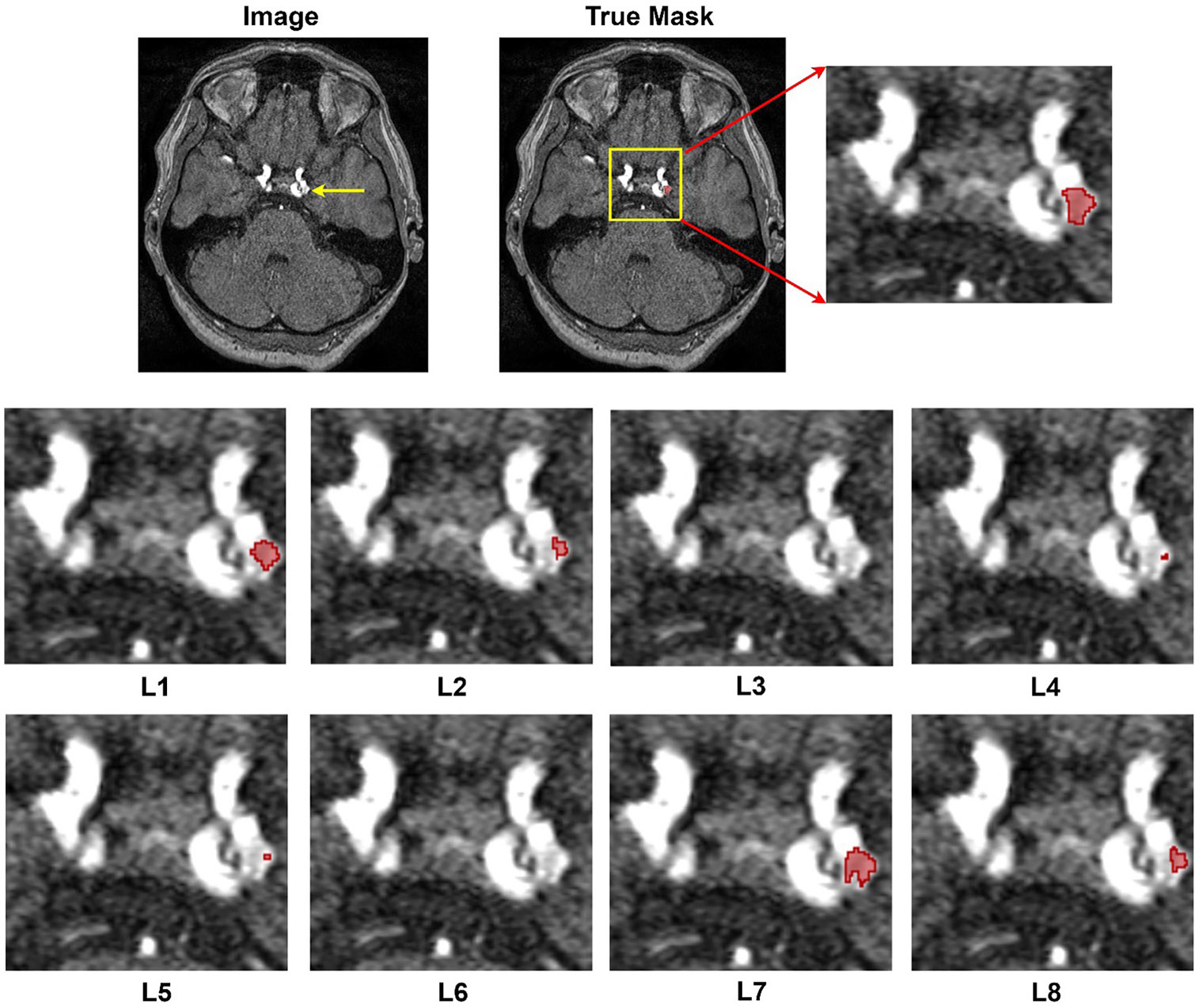
Segmentation results provided by different losses. Image#39 from ADAM dataset. L1: Dice + CE, L2: Dice + Top k, L3: Dice + CE + Top k, L4: Dice + Focal, L5: Dice + Top K + Focal, L6: Dice + Top k+ Focal + Tversky + CE, L7: Weighted Dice + Top k+ CE, and L8: Weighted Dice + Top k+ Focal + Tversky + CE.

The results from Table 4 showcase the outcomes derived from employing diverse loss functions in the training of nnU-Net for aneurysm segmentation using the RENJI dataset. The Weighted Dice + Top K + CE combination exhibits the highest mean Dice coefficient (0.7219), indicating superior overlap between predicted and ground truth regions. The Weighted Dice + CE + TopK combination achieves the highest sensitivity (0.6948), representing the model's ability to identify true positive instances. In terms of precision (0.8257), reflecting the correctness of positive predictions, Weighted Dice + CE + Top K obtains the highest mean value. For all metrics, the combination of Dice + CE secures the second rank. The variations in these metrics underscore the influence of different loss functions on the model's performance. Notably, Dice + Top k + CE and its weighted variant demonstrate the minimum SD for Dice and Sensitivity, while the combination of Dice and TopK yields the minimum variation in Precision. In summary, akin to the ADAM dataset, the highest performance across all metrics is achieved by combining weighted Dice + CE + TopK, with Dice + CE ranking second. Figure 4 shows the segmentation results of all loss functions on Case #39 from the ADAM dataset. This case was selected because it contains a relatively small aneurysm (~3.5 mm), which represents a challenging scenario for segmentation and thus highlights differences between loss functions.

To better quantify the variability and potential clinical significance of performance differences between loss functions, we computed 95% confidence intervals (CIs) for Dice, Sensitivity, and Precision from the five-fold cross-validation results. For example, our best-performing configuration (Weighted Dice + Top-k + CE) achieved a mean Dice score of 0.5467 (±0.019 SD), corresponding to an approximate 95%CI of 0.5467 ± 1.96×(0.019/√5) ≈ [0.529, 0.564]. In contrast, the baseline Dice + CE model yielded 0.5280 ± 0.031 SD, with a 95%CI of ≈ [0.501, 0.555]. The overlap of these CIs indicates that the observed absolute Dice improvement (~0.02) may not be statistically significant across folds, but the narrower interval for our proposed loss suggests greater stability. Similar patterns were observed for Sensitivity and Precision: the Weighted Dice + Top-k + CE model produced higher mean Sensitivity (0.5377 ± 0.021 SD; 95%CI ≈ [0.519, 0.556]) and Precision (0.7118 ± 0.020 SD; 95%CI ≈ [0.694, 0.729]) compared with the baseline. These intervals provide a clearer view of performance consistency and show that, while some differences are modest, our hybrid loss configuration consistently outperforms or matches other combinations with tighter variability, which is desirable for clinical translation.

Alongside the simulation outcomes presented in this study, we entered the Aneurysm Detection and Segmentation Challenge with our optimal loss function ( weighted Dice + CE + TopK). Our approach secured the fourth position in the segmentation task. Despite the challenge of assessing our model using external data, it achieved a Dice coefficient of 39%, with the top-ranking entry achieving 43% Dice.

#### Statistical analysis

This section employs the Kruskal-Wallis test to analyze the performance measure values presented in Tables 3 and 4. The Kruskal-Wallis test, as a non-parametric approach, is utilized to compare two or more independent samples of equal or varying sizes. A significant outcome from the Kruskal-Wallis test suggests that at least one sample stochastically dominates another.

Hence, this test was performed to determine whether a statistically significant difference (at the 95% confidence level) existed among the values derived from eight distinct loss functions for each performance metric in the ADAM and RENJI datasets. The *P*-values obtained from the Kruskal-Wallis tests indicate statistically significant differences in Dice, Sensitivity, and Precision values among all loss functions in the RENJI dataset. However, only Sensitivity and Precision show a significant difference in the ADAM dataset. In instances where the Kruskal-Wallis test *P*-value indicated a statistically significant difference, the Mann–Whitney U test was applied to determine which loss functions showed significant differences in performance metrics. This secondary test also operates under a 95% confidence level. Table 6 presents the outcomes of the Mann-Whitney U tests of the optimal loss (weighted Dice+ CE + TopK) for each dataset, with "−" indicating no statistically significant difference between specific performance metric values.

#### Cropping

To eliminate irrelevant information and define a Region of Interest (ROI), two strategies were implemented. First, we manually cropped images and labels by considering the location of aneurysms in the dataset, covering approximately 40% along the x-axis (left to right) and 50% along the y-axis (anterior to posterior). Second, an automatic vessel extraction approach was devised using the Hessian matrix, Otsu's method, and non-maximum suppression to enhance nnU-Net's performance and the images were cropped based on the vessel's location. In both scenarios, the nnU-Net was trained using the combination of Dice and Cross Entropy (CE) as the loss function. We performed both scenarios on the ADAM and RENJI datasets.

Table 7 provides the average and standard deviation of performance metrics when employing original data, manually cropped data, and vessel-based cropped data. The model was initially trained with original images of a 512 × 512 matrix, achieving 52.8%, 52.9%, and 68.9% for Dice, Sensitivity, and Precision, respectively. Subsequently, training with manually cropped images (reduced to 300 × 250) led to improved segmentation performance, reaching 53.6%, 53.2%, and 70.5%. It is noteworthy that employing a uniform scale for cropping resulted in varied effects on segmentation performance. While cropping enhanced performance in some cases, it led to the absence of aneurysms in cropped images, affecting overall model training. Vessels were then extracted for automatic cropping, resulting in a 59.8% reduction in the vessel-constrained images. The Dice coefficient increased to 56.1%, Sensitivity rose to 55.4%, and Precision increased to 73.4%. The nnU-Net was also analyzed for its performance with aneurysms of different sizes. For aneurysms larger than 3mm, using both the original and automatically cropped images, nnU-Net achieved 59.2% and 65.8% for Dice and 58.3% and 62.5% for Sensitivity, respectively. The Mann-Whitney U test revealed statistically significant differences between the nnU-Net trained on original data and vessel-based cropped data (*P* = 0.015 for Dice, 0.039 for Sensitivity, and 0.032 for Precision). However, these differences were small in magnitude, indicating that although they are statistically significant, they are unlikely to be clinically meaningful.

Concerning the RENJI dataset, the initial training of the model involved original images with a matrix size of 512 × 512, yielding percentages of 68.1%, 66.8%, and 79.3% for Dice, Sensitivity, and Precision, respectively. Subsequent training with manually reduced images (downsized to 300 × 250) resulted in enhanced segmentation performance, achieving 69.4%, 67.9%, and 81%. It is important to note that the application of a consistent scale for cropping led to diverse impacts on segmentation performance. While cropping improved performance in certain instances, it resulted in the absence of aneurysms in cropped images, influencing the overall training of the model. Vessels were then extracted for automated cropping, resulting in an average reduction of 55.3% in vessel-constrained images. The Dice coefficient increased to 72.3%, Sensitivity rose to 68.4%, and Precision increased to 83.5%. Similar to the ADAM dataset, the statistical analysis indicates no significant difference between results obtained from original data and cropped images. However, the Mann-Whitney U test unveiled a statistically significant difference, with *P*-values of 0.033 for Dice and 0.031 for Precision between nnU-Net trained with original data and vesselbased cropped data.

#### Multi-modality training

In this section, we employed two modalities for the training of nnU-Net. The first modality comprises the utilization of original 3D TOF-MRA images, while the second modality involves an attention-based approach. The attention-based image is generated through the multiplication of the original image with the normalized vessel-enhanced image obtained from the Hessian matrix.

This dual-modality training strategy aims to leverage both the inherent information present in the original images and the attention-enhanced features derived from the vascular structures, ultimately enhancing the nnU-Net's ability to capture important nuances in the data. In line with previous sections, we utilized nnU-Net trained with the Dice + CE loss function. Table 8 presents the segmentation outcomes obtained through single-modality training compared to multi-modality training. For the ADAM dataset, the adoption of multi-modality training resulted in a modest improvement, elevating the Dice score from 52.8% to 53.3%, increasing Sensitivity from 52.9% to 53.19%, and raising Precision from 68.9% to 69.1%. These increments, while slight, exhibit a discernible enhancement, yet the statistical analysis reveals no significant difference in the results. On the other hand, the improvement observed in the RENJI dataset is more substantial. Although there is no significant difference in the Dice results, the statistical analysis of Sensitivity and Precision indicates that there is a statistically significant difference when employing multi-modality for training nnU-Net.

## DISCUSSIONS

Our study investigated three key contributions to the segmentation of intracranial aneurysms using deep learning models and 3D TOF-MRA images: the impact of different hybrid loss functions, the effectiveness of training with cropped data based on vessel information, and the advantages of multi-modality training. Firstly, regarding the exploration of hybrid loss functions, our results demonstrate varying degrees of effectiveness in improving segmentation performance. The weighted combination of Dice, CE, and Top K loss function yielded the highest overall performance across all metrics in both ADAM and RENJI datasets. This finding suggests that a blend of distribution-based and region-based losses can effectively guide the model's attention and enhance segmentation accuracy. However, the choice of the optimal loss function may depend on specific priorities, such as emphasizing precision over sensitivity or achieving a balanced Dice coefficient. Secondly, our investigation into training with cropped data revealed promising results. Both manual cropping and automatic extraction based on vessel information showed improvements in segmentation performance compared to using original images. Particularly, the automatic vessel-based cropping approach demonstrated significant enhancements, with notable increases in Dice coefficient, Sensitivity, and Precision. However, it's essential to consider the potential trade-offs, as uniform cropping may lead to the exclusion of relevant information, affecting overall model training. Lastly, our study explored the benefits of multi-modality training, integrating original 3D TOF-MRA images with attention-enhanced features derived from vascular structures. While the improvement observed in the ADAM dataset was modest, the RENJI dataset showed more substantial enhancements in Sensitivity and Precision. The RENJI dataset contains more complex cases (including ruptured aneurysms, fusiform aneurysms, and a wider size range, with a median of 9.35 *vs.* 3.6 mm for ADAM). In such complex anatomical contexts, incorporating vessel-enhanced images as an 'attention mechanism' more effectively helps the model focus on critical vascular regions, thereby exerting a greater impact than in the relatively simple and highly homogeneous ADAM dataset.

There are a few limitations of this study. First, the sample size is relatively small, and the validation of our method in large-scale multi-center data will be required in the future. The ADAM dataset is biased toward small aneurysms (median 3.6 mm), while the RENJI dataset contains more large aneurysms (median 9.35 mm). This distribution bias (Dataset Bias) may lead to biased performance evaluation of the model in specific size ranges and limit the validation of the model's generalization ability across the full size spectrum (from tiny to giant aneurysms). Second, only 3D TOF MRA was used in this study. Other commonly used imaging modalities for intracranial aneurysms, including contrast-enhanced MRA (CE-MRA) and computed tomographic angiography (CTA), were not evaluated in this study. The proposed method needs to be validated in other image modalities in the future. The 3D TOF MRA is a widely used clinical protocol that is non-invasive, radiation-free, and does not require contrast, making it highly suitable for intracranial aneurysm screening and imaging surveillance. The application of our method will still cover a large population. Third, we recognize several practical challenges for clinical implementation. Robust deployment will require reproducible preprocessing and model training across institutions, careful validation on heterogeneous imaging protocols and scanners, and the development of secure frameworks for data sharing to support external benchmarking and continuous improvement. Addressing these issues, along with exploring additional loss functions, refining cropping strategies, and integrating a wider range of multi-modal data, represents an important direction for future work and will be essential for translating automated aneurysm segmentation into routine neuroradiological practice.

## CONCLUSIONS

This study refined the nnU-Net pipeline for accurate intracranial aneurysm segmentation using 3D TOF-MRA images. Among the tested configurations, the weighted Dice + CE + TopK loss function − followed closely by Dice + CE − yielded the highest segmentation performance. Incorporating vessel information via automatic segmentation improved the model’s focus on aneurysm-prone regions, and adopting multi-modality training that combined original and vessel-enhanced images produced significant gains in Sensitivity and Precision on the RENJI dataset. These results highlight the robustness and practical potential of our approach.

## Figures and Tables

**Table 1.** Dataset characteristics

Dataset	Number	Size			Dark signal	Multiple aneurysms	Shape	
		< 5	[5, 10]	> 10			Fusiform	Saccular
RENJI	213	12	152	49	108	25	55	198
ADAM	89	45	43	1	1	27	88	2

**Table 2.** Loss functions explanation

Loss	Expression	Note
CE	$-y_{i}log{\hat{y}}_{i}-(1-y_{i})\log\;(1-{\hat{y}}_{i})$	*y* → *grounf truth*,ŷ → *prediction*
Dice	$1-\frac{2\sum_{i}y_{i}{\hat{y}}_{i}}{\sum_{i}({y_{i}}^{2}+{{\hat{y}}_{i}}^{2})}$	
TopK	$-\frac{\sum_{c=1}^{C}\sum_{i=1}^{N}1\{y_{i}=c\;and\;{\hat{y}}_{i}^{c}<t\}\;\log\;{\hat{y}}_{i}^{c}}{\sum_{c=1}^{C}\sum_{i=1}^{N}1\{y_{i}=c\;and\;{\hat{y}}_{i}^{c}<t\}}$	*t* → *threshold*, 1 → *binary indicator function*
Focal	$-(\alpha {\left(1-{\hat{y}}_{i}\right)}^{\gamma }y_{i}log{\hat{y}}_{i}+\;(1+\alpha ){{\hat{y}}_{i}}^{2}(1-y)\;\log(1-{\hat{y}}_{i}))$	
Tversky	$\frac{\begin{array}{c} \sum_{c=1}^{C}1-TI,\\ \text{Tversky index}\;(TI)=\\ \sum_{i=1}^{N}p_{0i}g_{0i} \end{array}}{\sum_{i=1}^{N}p_{0i}g_{0i}+\alpha \sum_{i=1}^{N}p_{0i}g_{1i}+\beta \sum_{i=1}^{N}p_{1i}g_{0i}}$	*p*_0*i*_: *probability of pixel “I” belonging to the foreground class* *p*_1*i*_: *probability of pixel belonging to the background class* *g*_0*i*_:*foreground:1 & background:0* *g*_0*i*_: *foreground:0 & background:1*

**Table 3.** The mean and standard deviation of performance metrics for various loss functions in nnU-net on ADAM dataset

Metric	Dice + CE	Dice + Top k	Dice + CE + Top k	Dice + Focal	Dice + Top K + Focal	Dice + Top k+ Focal + Tversky + CE	Weighted Dice + Top k+ CE	Weighted Dice + Top k+ Focal + Tversky + CE
dice	0.5280 ± 0.031	0.5083 ± 0.045	0.5003 ± 0.023	0.5044 ± 0.018	0.4896 ± 0.043	0.4827 ± 0.026	**0.5467 ± 0.019**	0.5181 ± 0.033
sensitivity	0.5298 ± 0.027	0.4887 ± 0.052	0.4884 ± 0.040	0.4999 ± 0.022	0.4661 ± 0.056	0.4772 ± 0.029	**0.5377 ± 0.021**	0.5102 ± 0.043
precision	0.6896 ± 0.020	0.681 ± 0.013	0.6702 ± 0.042	0.6632 ± 0.044	0.6867 ± 0.033	0.6529 ± 0.027	**0.7118 ± 0.020**	0.6911 ± 0.024

Bold numbers: The highest value.

**Table 4.** The mean and standard deviation of performance metrics for various loss functions in nnU-net on RENJI dataset

Metric	Dice + CE	Dice + Top k	Dice + CE + Top k	Dice + Focal	Dice + Top K + Focal	Dice + Top k+ Focal + Tversky + CE	Weighted Dice + Top k+ CE	Weighted Dice + Top k+ Focal + Tversky + CE
Dice	0.6818 ± 0.027	0.5727 ± 0.041	0.5650 ± 0.020	0.5711 ± 0.027	0.5378 ± 0.049	0.5019 ± 0.029	**0.7219 ± 0.024**	0.5722 ± 0.036
Sensitivity	0.6685 ± 0.022	0.5228 ± 0.057	0.5318 ± 0.037	0.5028 ± 0.029	0.4851 ± 0.049	0.4823 ± 0.022	**0.6948 ± 0.022**	0.5236 ± 0.048
Precision	0.7936 ± 0.029	0.6618 ± 0.013	0.6936 ± 0.045	0.7018 ± 0.047	0.6639 ± 0.030	0.6419 ± 0.019	**0.8257 ± 0.019**	0.6708 ± 0.015

Bold numbers: The highest value.

**Table 5.** The performance of the proposed method in the ADAM challenge and the rank

	DSC	MHD	VS
Average	0.39	14.80	0.50
Rank	0.097	0.098	0.181

DSC: Dice similarity coefficient; MHD: Hausdorff distance (modified, 95th percentile); VS: volumetric similarity; (Ref: The ADAM Challenge’s website).

**Table 6.** Results of Mann-Whitney U test. L1: Dice + CE, L2: Dice + Top k, L3: Dice + CE + Top k, L4: Dice + Focal, L5: Dice + Top K + Focal, L6: Dice + Top k+ Focal + Tversky + CE, L7: Weighted Dice + Top k+ CE, and L8: Weighted Dice + Top k+ Focal + Tversky + CE

Dataset	Metric	Loss	L1	L2	L3	L4	L5	L6	L8
ADAM	Dice	L7	-	1.2E-2	3.5E-2	4.4E-2	6.9E-3	6.5E-3	3.1E-2
	Sensitivity	L7	-	1.1E-2	1.1E-2	4.2E-2	8.2E-3	5.7E-3	3.3E-2
	Precision	L7	3.9E-2	4.6E-2	3.3E-2	8.6E-3	3.5E-2	3.5E-2	4.5E-2
RENJI	Dice	L7	1.5E-2	4.6E-4	5.1E-4	4.9E-4	5.1E-3	5.5E-4	6.6E-4
	Sensitivity	L7	2.5E-2	4.9E-3	2.3E-3	3.3E-4	4.3E-3	3.2E-4	6.9E-4
	Precision	L7	2.9E-2	6.3E-4	4.7E-2	6.2E-3	7.1E-3	3.5E-3	6.1E-4

**Table 7.** The Average and Standard Deviation of Performance Metrics when employing original data, manually cropped data, and vessel-based cropped data in nnU-Net on the ADAM and RENJI Dataset

Dataset	Metric	Trained by original data	Trained by manually cropped data	Trained by vessel-based cropped data
ADAM	Dice	0.5280 ± 0.031	0.5360 ± 0.039	0.5618 ± 0.026
	Sensitivity	0.5298 ± 0.027	0.5325 ± 0.047	0.5543 ± 0.039
	Precision	0.6896 ± 0.020	0.7055 ± 0.028	0.7347 ± 0.035
RENJI	Dice	0.6818 ± 0.027	0.6948 ± 0.032	0.7236 ± 0.033
	Sensitivity	0.6685 ± 0.022	0.6793 ± 0.040	0.6841 ± 0.037
	Precision	0.7936 ± 0.029	0.8105 ± 0.037	0.8355 ± 0.035

**Table 8.** The segmentation comparison of single-modality *vs.* multi-modality training in nnU-Net

Dataset	Metric	Single-modality training	Multi-modality training	*P*-value
ADAM	Dice	0.5280 ± 0.031	0.5333 ± 0.031	0.41
	Sensitivity	0.5298 ± 0.027	0.5319 ± 0.035	0.62
	Precision	0.6896 ± 0.020	0.6917 ± 0.027	0.55
RENJI	Dice	0.6818 ± 0.027	0.7022 ± 0.033	0.08
	Sensitivity	0.6685 ± 0.022	0.6935 ± 0.039	0.012
	Precision	0.7936 ± 0.029	0.8318 ± 0.025	0.019
